# Transcriptional positive cofactor 4 promotes breast cancer proliferation and metastasis through c-Myc mediated Warburg effect

**DOI:** 10.1186/s12964-019-0348-0

**Published:** 2019-04-16

**Authors:** Peng Luo, Chi Zhang, Fengying Liao, Long Chen, Zhenyu Liu, Lei Long, Zhongyong Jiang, Yawei Wang, Ziwen Wang, Zujuan Liu, Hongming Miao, Chunmeng Shi

**Affiliations:** 1 Institute of Rocket Force Medicine, State Key Laboratory of Trauma, Burns and Combined Injury, Third Military Medical University, Chongqing, 400038 China; 2 Institute of Immunology, Third Military Medical University, Chongqing, 400038 China; 3 Department of Biochemistry and Molecular Biology, Third Military Medical University, Chongqing, 400038 China

**Keywords:** Breast cancer, PC4, Warburg effect, C-Myc, Metastasis, Proliferation

## Abstract

**Background:**

The human positive cofactor 4 (PC4) is initially identified as a transcriptional cofactor and has an important role in embryonic development and malignant transformation. However, the clinical significance and the molecular mechanisms of PC4 in breast cancer development and progression are still unknown.

**Methods:**

We investigated PC4 expression in 114 cases of primary breast cancer and matched normal breast tissue specimens, and studied the impact of PC4 expression as well as the molecular mechanisms of this altered expression on breast cancer growth and metastasis both in vitro and in vivo.

**Results:**

PC4 was significantly upregulated in breast cancer and high PC4 expression was positively correlated with metastasis and poor prognosis of patients. Gene set enrichment analysis (GSEA) demonstrated that the gene sets of cell proliferation and Epithelial-Mesenchymal Transition (EMT) were positively correlated with elevated PC4 expression. Consistently, loss of PC4 markedly inhibited the growth and metastasis of breast cancer both in vitro and in vivo. Mechanistically, PC4 exerted its oncogenic functions by directly binding to c-Myc promoters and inducing Warburg effect.

**Conclusions:**

Our study reveals for the first time that PC4 promotes breast cancer progression by directly regulating c-Myc transcription to promote Warburg effect, implying a novel therapeutic target for breast cancer.

**Electronic supplementary material:**

The online version of this article (10.1186/s12964-019-0348-0) contains supplementary material, which is available to authorized users.

## Background

Breast cancer is one of the most common cancers and a leading cause of tumor-related death in females worldwide. Despite advances in treatments, many patients still develop into metastatic disease, which is the leading cause of breast cancer death [1–3]. Thus, there is an urgent need to characterize the underlying molecular mechanisms and identify novel therapeutic targets to improve the outcomes for breast cancer [4].

Recent studies have shown that metabolic reprogramming is a hallmark of cancer cells and a key contributor to cancer progression [5]. The Warburg effect [6], as known as aerobic glycolysis, is the best-characterized metabolic change in cancer cells, which facilitates cancer growth and progression by increasing glucose uptake, elevating lactate production, and supporting the energy demands [7]. Emerging evidence has indicated the crucial role of the Warburg effect in cancer therapy as a novel target [8]. In the process of glycolysis, the c-Myc oncogenic transcription factor plays a key role by directly regulating the glycolytic genes, such as GLUT1, HK2, LDHA, PDK1, etc. [9, 10]. Although a few transcription factors have been reported to regulate glycolysis [11], the transcriptional regulation of Warburg effect and the upstream regulatory mechanism of c-Myc are still largely unknown.

The human positive cofactor 4 (PC4) and its yeast ortholog SUB1 (also named as coactivator p15) are initially identified as a coactivator of basal transcription [12, 13]. PC4 is located on chromosome 5p13 and encodes a 127-amino acid protein that has an important role in various cellular processes including transcription [14–17], DNA replication [18–22], DNA repair [23–31] and chromatin organization [32, 33]. By the model of PC4 knockout mouse, we have found that loss of PC4 results in early embryo lethality, highlighting an essential role of PC4 in embryonic development [34]. And our previous studies have shown that overexpression of PC4 is involved in the malignant transformation of normal dermal multipotent fibroblasts, indicating the crucial role of PC4 in tumorigenesis [35]. Besides, PC4 is also found to be upregulated in lung cancer [36], astrocytoma [37], prostate cancer [38] and esophageal squmaous cell carcinoma [26] and positively related with tumor lymphatic metastasis [39] and chemo-radiosensitivity [26, 40, 41]. However, the clinical significance and the molecular mechanisms of PC4 in breast cancer development and progression are still unknown.

In this study, we reported firstly that PC4 was highly expressed in breast cancer and positively correlated with metastasis and poor prognosis of patients. Through gene set enrichment analysis (GSEA) in breast cancer specimens and experimental verification, we demonstrated that PC4 promoted the growth and metastasis of breast cancer both in vitro and in vivo. Furthermore, our findings revealed that PC4 exerted its oncogenic functions by directly binding to c-Myc promoters and activating glycolysis. Therefore, our study provided novel insights into the functions and mechanisms of PC4 in breast cancer, and suggested that PC4 might be a promising therapeutic target for breast cancer.

## Methods

### Cell lines and clinical samples

The human breast cancer cell lines (MDA-MB-231 and MCF-7) were purchased from the American Type Culture Collection (ATCC, USA) and the Cell Bank of the Chinese (Shanghai, China). All cells were cultured in the recommended medium (Hyclone, USA), supplemented with 10% FBS (Gibco, USA) and 1% streptomycin/penicillin (Beyotime, Shanghai, China), and incubated in 5% CO_2_ at 37 °C. A total of 114 paraffin-embedded breast cancer samples, with paired adjacent tissue and normal tissues were obtained at Southwest Hospital of Third Military Medical University. All breast cancer patients were diagnosed independently by at least two experienced pathologists, according to the Union for International Cancer Control classification system. The study was approved by the Ethics Committee of Third Military Medical University.

### Cell proliferation assay and colony formation assay

Cell proliferation was measured with the Cell Counting Kit-8 (Dojindo, Japan). Cells were seeded into 96-well plates with a density of 2000 cells each well and 100 ul medium. Cellular proliferation was measured according to the readout at the wavelength of 450 nm. Data were read by a microplate reader (Multiskan Go Multimode Reader; Thermo Scientific). To determine their clonogenic ability, MDA-MB-231 and MCF-7 cells were trypsinized and seeded in 6-well plates. The medium was changed every three days, and cells were cultured for up to 14 day until colonies were clearly visible. At the endpoint, cells were washed twice with PBS, fixed with 4% paraformaldehyde, stained with crystal violet (Beyotime, China) for 30 min, and then colonies with > 50 cells were counted.

### Wound healing assay

After transfection with the respective shRNAs, cells were tripsinized and seeded into 6-well plates to allow the cells to form a complete monolayer. After they became confluent, wounds were carefully introduced across the cell monolayer with a 10ul pipette tip. The dead cells were removed by PBS washes. Cells were treated with serum-free medium for 24 h. Images were taken at the indicated time and then migration distance was measured.

### Transwell chamber assay

Cell inserts pre-coated with matrigel (8.0 μm pore size membrane; Corning) were used. Breast cancer cells were deprived of serum for 12 h and seeded onto the upper chamber surface in serum-free medium at a density of 1 × 10^5^ cells per well, and 20% FBS was placed in the lower chamber and incubated at 37 °C for 24 h. At the end of incubation, nonmotile cells on the upper surface of the filter were wiped off and the cells on the underside chamber were fixed with methanol and stained with Crystal Violet (Beyotime) and counted using bright-field microscopy in 5 random fields in triplicate inserts.

### ECAR, glucose uptake, lactate and ATP assays

Extracellular acidification rate (ECAR) was measured using extracellular flux analyzer (XFp) analyzer (Seahorse Bioscience) as described by the manufacturer. Lactate production (Lactate Assay Kit) was measured as described by the manufacturer (BioVision). To measure glucose uptake, cells were incubated with a fluorescent D-glucose derivative, 2-[N-(7-nitrobenz-2-oxa-1,3-diazol-4-yl)amino] -2-deoxy-D-glucose (2-NBDG; APExBIO) for 20 min at 37 °C. The fluorescence intensity of 2-NBDG was measured using by flow cytometry (BD FACSCanto II™), and data were analyzed with FlowJo software (Treestar). ATP production (Enhanced ATP Assay Kit) was measured according to the protocol recommended by manufacturer (Beyotime).

### RNA interference

The shRNA targeting human PC4(shRNA1: 5′- GACAGGUGAGACUUCGAGATT -3′; 5′- UCUCGAAGUCUCACCUGUCTT -3′. shRNA2: 5′- ACAGAGCAGCAGCAGCAGATT -3′; 5′- UCUGCUGCUGCUGCUCUGUTT -3′) and negative control shRNA (5′-UUCUCCGAACGUGUCACGUTT-3′; 5′-ACGUGACACGUUCGGAGAATT-3′) were constructed by GenePharma (Shanghai, China). The human PC4 plasmid and control were purchased from GeneChem (Shanghai, China). The human c-Myc plasmid and control were purchased from GenePharma (Shanghai, China). According to the manufacturer’s protocol, MDA-MD-231and MCF-7 cells were transfected with shRNA or plasmid according to the manufactures’ instructions.

### Western blotting analysis

MDA-MB-231 and MCF-7 cells were harvested, washed, and lysed with RIPA buffer (Beyotime, China) containing protease inhibitor cocktail (Roche) for 30 min on ice. Total protein was extracted, and quantitated by a BCA kit (Beyotime, China) according to the manufacturer’s instruction. The protein samples were separated by electrophoresis in 10%~ 12% gel, and then transferred onto PVDF membranes (Millipore). Blotted membranes were blocked and incubation with primary antibodies overnight at 4 °C. The membranes were washed 5 min for 3 times with TBST, and subsequently incubated 1 h with HRP-linked secondary antibody (Cell Signaling Technology, USA) at room temperature. The band intensities were visualized and detected by an enhanced chemiluminescence detection system (Bio-Rad Laboratories). Primary antibodies against c-Myc, E-cadherin, N-cadherin, Vimentin, GLUT1, LDHA and β-actin were obtained from Cell Signaling Technology. Primary antibodies against PC4 were obtained from Sigma.

### Quantitative RT-PCR

Total RNA was extracted from breast cancer cells using Trizol (Cwbiotech, China). 1 μg RNA was reverse transcribed into cDNA using the RevertAid First Strand cDNA Synthesis kit (#K1622, Thermo Fisher Scientific,Inc.) according to the manufacturer’s protocol. As described in our previous work, qRT-PCR was performed using a SYBR Green qPCR master mix (Takara) according to the manufacturer’s protocol. After the reactions were completed, the comparative threshold cycle (Ct) method was used to calculate the relative gene expression. GAPDH expression was used as the internal control. Human-specific primers were synthesized, and their sequences are shown in Additional file 1: Table S1.

### Chromatin immunoprecipitation (ChIP)

MDA-MB-231 cells were conducted ChIP assay with the SimpleChIP Enzymatic Chromatin IP Kit (Magnetic Beads) (#9003; Cell Signaling Technology) according to the manufacturer’s instructions. Chromatin fragments were immunoprecipitated with anti-PC4 (NB10059774; Novus Biologicals), or rabbit IgG (#2729; Cell Signaling Technology) coupled with ChIP Grade Protein G Magnetic Beads (#9006; Cell Signaling Technology). After DNA purification, quantitative PCR was performed using primers (Additional file 1: Table S2). The fold enrichment was calculated by normalizing samples of anti-TCF-1 or p-STAT3 to normal rabbit IgG controls.

### Tumorigenicity assays

Tumor model in vivo was established using 4-week-old female athymic nude mice from the Laboratory Animal Center of the Third Military Medical University (Chongqing, China) in a specific pathogen-free condition. Animal protocols were followed the Animal Care and Use Committee Guidelines of the Third Military Medical University. Mice were inoculated subcutaneously with 3 × 10^6^ respective MDA-MB-231 cells in 100 ul PBS at one dorsal site. Tumor growth was grossly monitored and measured with sliding calipers every 2 days. Volume of tumors were calculated according to the formula: volume (mm^3^) = (width^2^ X length)/2. When mice were killed, tumors were dissected and weighed. For the growth test, mice were sacrificed at the endpoint.

### Experimental metastasis assay

Tumor metastasis assays were performed using an intravenous breast cancer mouse model as previous described [42]. Briefly, respective MDA-MB-231 cells were digested in PBS. Single-cell suspensions of 3 × 10^5^ cells were injected into the tail vein of female NOD/SCID mice. Four weeks after injection, animals were sacrificed, lungs collected, and fixed in 10% buffered formalin for subsequent hematoxylin and eosin (H&E) staining.

### Immunohistochemical staining

The paraffin-embedded sections were dewaxed, rehydrated and incubated with human PC4 antibody (1: 500; Sigma, St. Louis, Missouri, USA) at 4 °C overnight. Subsequently, the slides were sequentially incubated with biotinylated secondary antibody at 37 °C for 30 min, and positive staining was visualized by using DAB. PC4 expression in breast cancer was evaluated by percentage of positive-staining cells and staining intensity. The percentage of positive cells was evaluated quantitatively and scored as: 0 (< 5% positive tumor cells), 1 (5–25% positive tumor cells), 2 (26–50% positive tumor cells), 3 (51–75% positive tumor cells) and 4 (> 75% positive tumor cells). Intensity was graded as follows: 0, no signal; 1, weak (light yellow); 2, moderate (brown); and 3, strong staining. The final quantification of each staining was obtained by multiplying these 2 scores. A total staining score of 0–12 was calculated and graded as negative (−, score 0–1), weak (+, score 2–4), moderate (++, score 5–8), or strong (+++, score 9–12). All tissue samples were examined and independently evaluated by two pathologists.

### Statistical analysis

Statistical analysis was carried out using SPSS 13.0 software (SPSS Inc., Chicago, USA), and all data are presented as means ± SD. Comparisons between two groups were performed using the Student’s t-test. Comparisons among three or more groups were performed using a one-way analysis of variance (ANOVA). The survival data was performed using the Kaplan-Meier method. Correlation between PC4 expression and clinical parameters was determined using the Pearson’s χ^2^ method. *P* < 0.05 indicated a statistically significant difference.

## Results

### PC4 is significantly upregulated in breast cancer and is closely correlated with metastasis and poor prognosis of patients

**Fig. 1:**
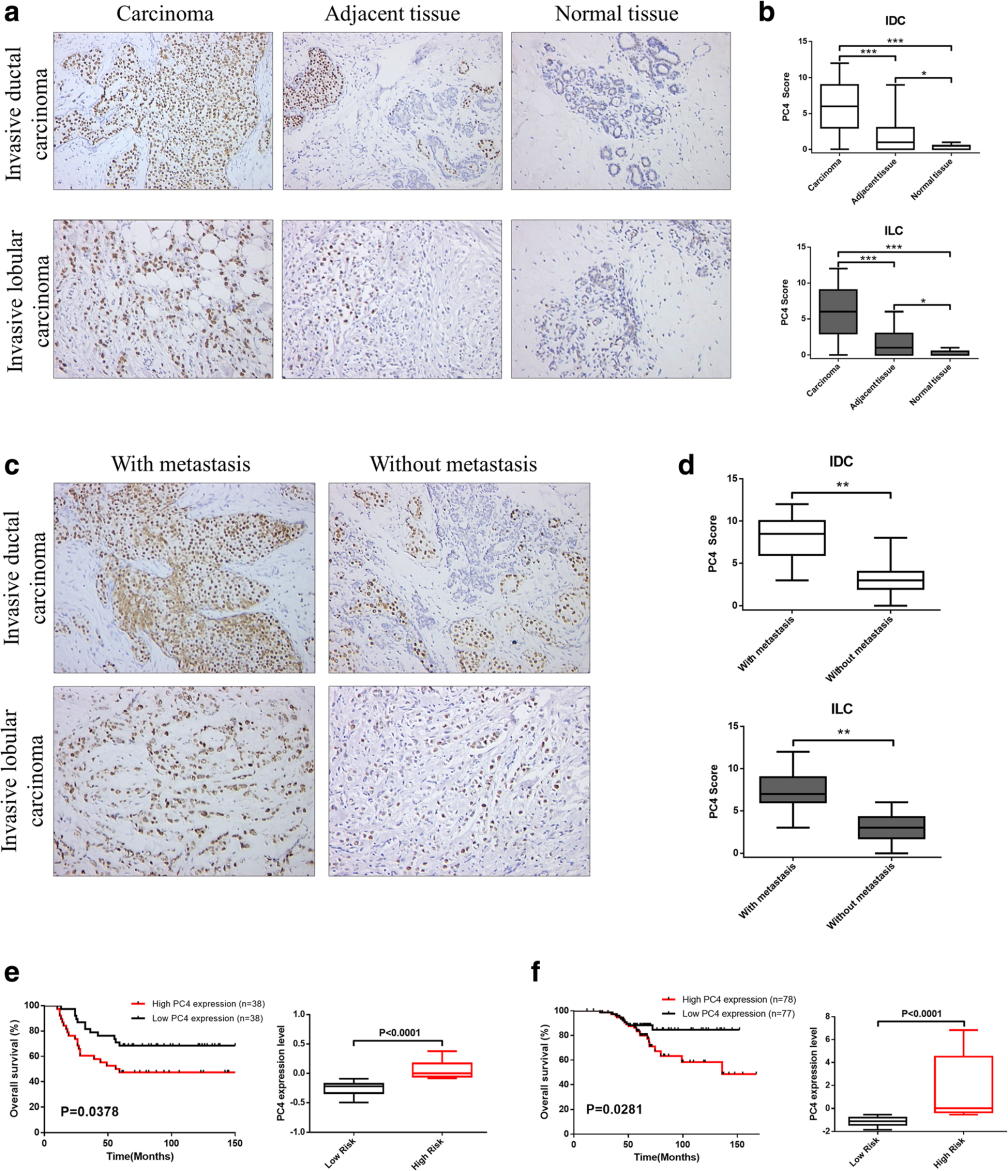
PC4 is upregulated in breast cancer and correlated with metastasis and poor prognosis of patients. **a** Immunohistochemical staining for PC4 protein in 114 cases of breast cancer (IDC and ILC) tissues, adjacent tissues and normal tissues. **b** The average staining score of PC4 expression in IDC and ILC derived from (a). **c** Immunohistochemical staining for PC4 protein in breast cancer (IDC and ILC) tissues with or without metastasis. **d** The average staining score of PC4 expression in IDC and ILC derived from (c). **e** Statistical analysis of the correlation between PC4 expression levels and overall survival in breast cancer. Data was obtained using Vant Veer breast cancer databases. **f** Kaplan-Meier analysis for the association of PC4 expression levels with overall survival time in breast cancer patients. Data was obtained from GSE9893. All data indicate the mean ± SD, *p < 0.05, **p < 0.01, ***p < 0.001

To explore the potential clinical significance of PC4, we firstly detected PC4 expression level in 114 cases of breast cancer patients compared with their adjacent counterparts. In both invasive ductal carcinoma (IDC) and invasive lobular carcinoma (ILC), aberrant PC4 overexpression was observed in carcinoma tissues from immunohistochemistry results, while weak positive signal was found in adjacent tissues and almost no positive signal in normal tissues (Fig. 1a). The average staining score of PC4 expression confirmed above results (Fig. 1b), implying a potential role of PC4 in breast cancer tumorigenesis. In addition, we analyzed PC4 expression in breast cancer with or without metastasis. As shown in Fig. 1c and d, the intensity of PC4 in carcinoma with metastasis was apparently higher than that without metastasis, suggesting a possible correlation between PC4 expression levels and progression of metastasis in breast cancer.

Meanwhile, through analyzing 3951 cases of breast cancer patients from public cancer databases (Kaplan–Meier plotter database), the higher PC4 expression group (*n* = 1976) had poorer overall survival compared with lower PC4 expression group (*n* = 1975) (*P* < 0.0001, Additional file 1: Figure S1). Data from Vant Veer breast cancer databases [43] and GSE9893 [44] also demonstrated that overall survival periods were shorter among patients with higher PC4 levels in the tumor (*P* < 0.05, Fig. 1e-f). Taken together, these results indicate that PC4 is commonly upregulated in breast cancer and may represent a significant molecular feature in breast cancer pathogenesis.

### PC4 knockdown suppresses breast cancer cell growth both in vitro and in vivo

**Fig. 2:**
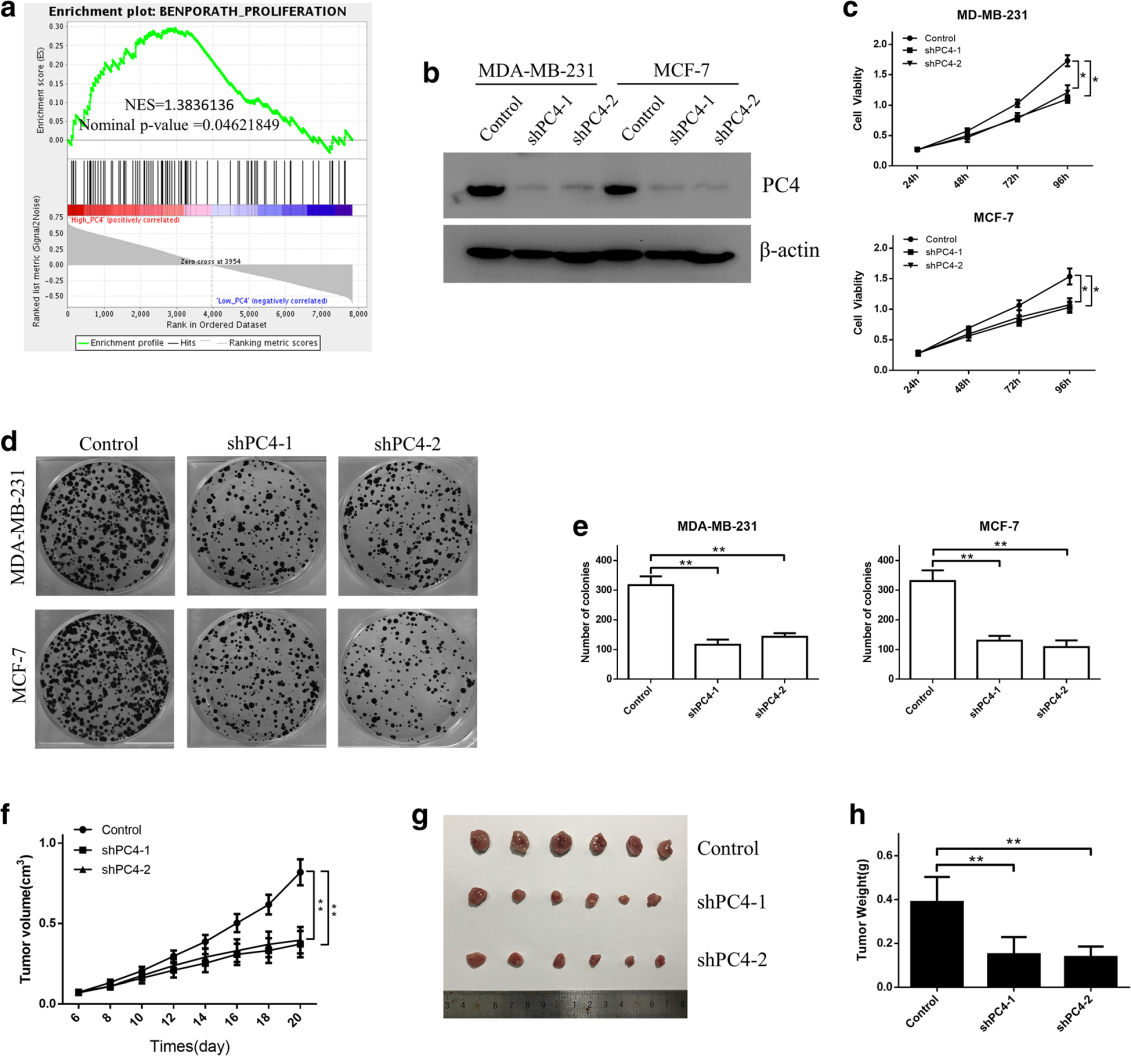
PC4 knockdown suppresses breast cancer cell growth both in vitro and in vivo. **a** Gene Set Enrichment Analysis (GSEA) comparing the gene sets of Proliferation in PC4^high^ (*n* = 78) and PC4^low^ (*n* = 77) breast cancer patients. Data was obtained from GSE9893. NES means normalized enrichment score. **b** Two shRNAs (shPC4–1 and shPC4–2) were used to establish the stable PC4 knockdown cell lines (MDA-MB-231 and MCF-7 cells). The PC4 knockout efficiency were examined by western blot. **c** Cell viability of MDA-MB-231 and MCF-7 cells was measured using CCK-8 assay at 24 h, 48 h, 72 h and 96 h after PC4 knockdown. **d** The clone formation assay of MDA-MB-231 and MCF-7 cells after PC4 knockdown. **e** Statistical analysis of the data derived from (d). Experiments were repeated three times independently. **f** MDA-MB-231 cells with stable PC4-knockdown were inoculated into female nude mice. Tumor growth curves were measured every 2 days, and the tumor volume was estimated using the following formula: volume = length×width^2^/2. **g** and **h** The dissected xenografts were photographed and weighed at the endpoint. All data indicate the mean ± SD. *p < 0.05, **p < 0.01

To investigate the functional significance of increased PC4 expression in breast cancer, we conducted gene set enrichment analysis (GSEA) to compare the gene expression profiles of PC4^low^ and PC4^high^ breast cancer specimens. GSE9893 dataset [44] containing 155 cases of breast cancer patients was divided into PC4^low^ (*n* = 78) and PC4^high^ (*n* = 77) groups based on the median expression level of PC4. GSEA results demonstrated that the gene sets of Benporath_Proliferation were obviously enriched in PC4^high^ breast cancer specimens, implying the potential role of PC4 in tumor proliferation (P < 0.05, Fig. 2a). To verify the above results, MDA-MB-231 and MCF-7 cells were chosen for subsequent loss-of-function studies. The stable cell lines with PC4 knockdown were established by lentiviral infection (shPC4–1 and shPC4–2) (Fig. 2b). The CCK-8 and colony formation assays demonstrated that silencing of PC4 dramatically reduced the proliferation (Fig. 2c) and colony formation capacity (Fig. 2d-e) of breast cancer cells. Besides, silencing of PC4 had no significant impact on cell apoptosis in both MDA-MB-231 and MCF-7 cells (Additional file 1: Figure S2).

Furthermore, we established a subcutaneous xenograft model to determine the effect of PC4 on breast cancer cell growth in vivo. The MDA-MB-231 cells with or without stable PC4-knockdown were inoculated subcutaneously into the right flank of female nude mice, and tumor sizes were measured every 2 days. During the whole experiment, the growth of xenografts in shPC4–1 and shPC4–2 group was inhibited compared with the control group (Fig. 2f). And the average tumor size and tumor weight at the endpoint was reduced by PC4 depletion (Fig. 2g-h). Taken together, these data suggest that PC4 promotes breast cancer cell growth both in vitro and in vivo.

### Loss of PC4 inhibits metastasis of breast cancer via reducing epithelial-mesenchymal transition both in vitro and in vivo

**Fig. 3:**
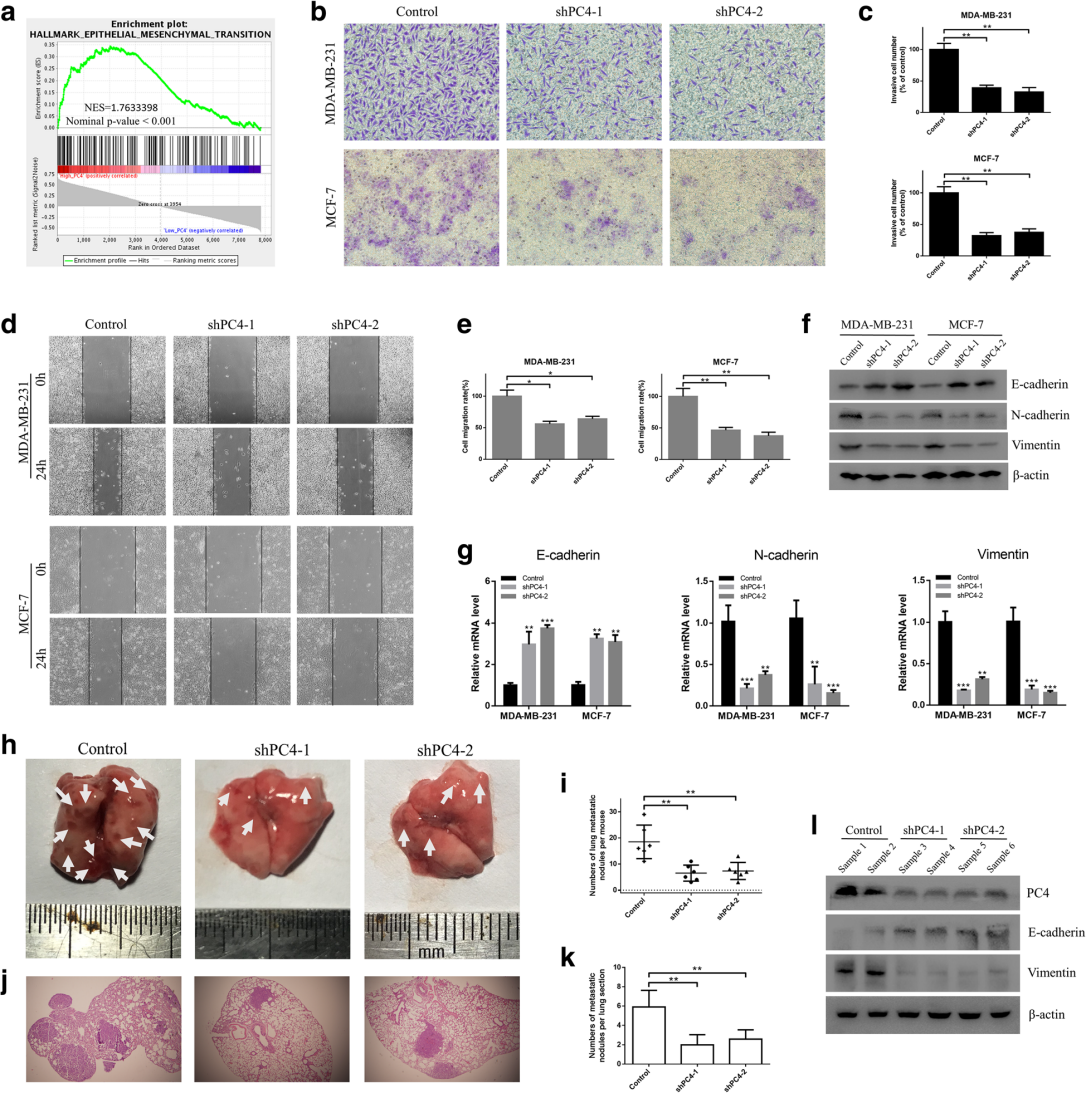
Loss of PC4 inhibits metastasis of breast cancer cells in vitro and in vivo. **a** GSEA comparing the gene sets of Epithelial-Mesenchymal Transition (EMT) in PC4^high^ (*n* = 78) and PC4^low^ (*n* = 77) breast cancer patients. Data were obtained from GSE9893. NES means normalized enrichment score. **b** Cell invasive capacity was detected using the transwell assay after PC4 knockdown in MDA-MB-231 and MCF-7 cells. **c** Statistical analysis of the data derived from (c). Experiments were repeated three times independently. **d** Cell migration capacity was measured using the wound healing assay after PC4 knockdown in MDA-MB-231 and MCF-7 cells. **e** Statistical analysis of the data derived from (d)**.** Experiments were repeated three times independently. **f** EMT markers (E-cadherin, N-cadherin and Vimentin) were detected by western blotting in MDA-MB-231 and MCF-7 cells after PC4 knockdown. **g** The mRNA levels of EMT markers (E-cadherin, N-cadherin and Vimentin) were detected by qPCR in MDA-MB-231 and MCF-7 cells after PC4 knockdown. **h** Tail vein injection of PC4-knockdown and control MDA-MB-231 cells in NOD/SCID mice were used to establish lung metastasis model. Lung tissue was dissected and photographed at the endpoint. **i** Statistical analysis of lung metastatic lesions per mouse in PC4 knockdown groups and control group. **j** Hematoxylin and eosin (H&E) staining of lung tissue in PC4 knockdown groups and control group. **k** Statistical analysis of lung metastatic nodules per lung section in PC4 knockdown groups and control group. **l** EMT markers (E-cadherin and Vimentin) were detected by western blot in dissected xenografts. All data indicate the mean ± SD, *p < 0.05, **p < 0.01, ***p < 0.001.

Apart from the gene sets of proliferation, GSEA in GSE9893 also showed that the gene sets of HALLMARK_Epithelial_Mesenchymal_Transition were significantly enriched in PC4^high^ breast cancer samples (*P* < 0.001, Fig. 3a). Owing to the crucial role of EMT in tumor metastasis [45], we conducted further experiments in vitro. The wound healing assays and transwell assays revealed that depletion of PC4 led to a significant inhibition of migration (Fig. 3d-e) and invasive capability (Fig. 3-c) in both MDA-MB-231 and MCF-7 cells. Then, we detected the expression levels of EMT markers by western blot and qPCR. As shown in Fig. 3f and g, the protein and mRNA level of epithelial markers such as E-cadherin were increased, while the protein level of mesenchymal markers such as N-cadherin and Vimentin were reduced in PC4-knockdown cells. These results demonstrate that PC4 can promote cell migration and invasion and induce EMT in breast cancer cells.

Next, lung metastasis model, which was established by tail vein injection of PC4-knockdown and control breast cancer cells, was used to evaluate the role of PC4 in tumor metastasis in vivo. Female NOD/SCID mice injected with PC4-knockdown MDA-MB-231 cells showed a significant decrease of lung metastatic lesions in comparison with control group (Fig. 3 h-i). And the hematoxylin and eosin (H&E) staining of lung tissues confirmed that depletion of PC4 resulted in greatly decreased lung metastasis of breast cancer cells in vivo (Fig. 3j-k). Moreover, Fig. 3 l showed that downregulation of PC4 decreased the expression of Vimentin and increased the expression of E-cadherin, indicating that PC4 could induce EMT in vivo. Collectively, PC4 plays an important role in breast cancer metastasis via inducing EMT.

### Glycolysis is critically involved in the oncogenic functions of PC4

**Fig. 4:**
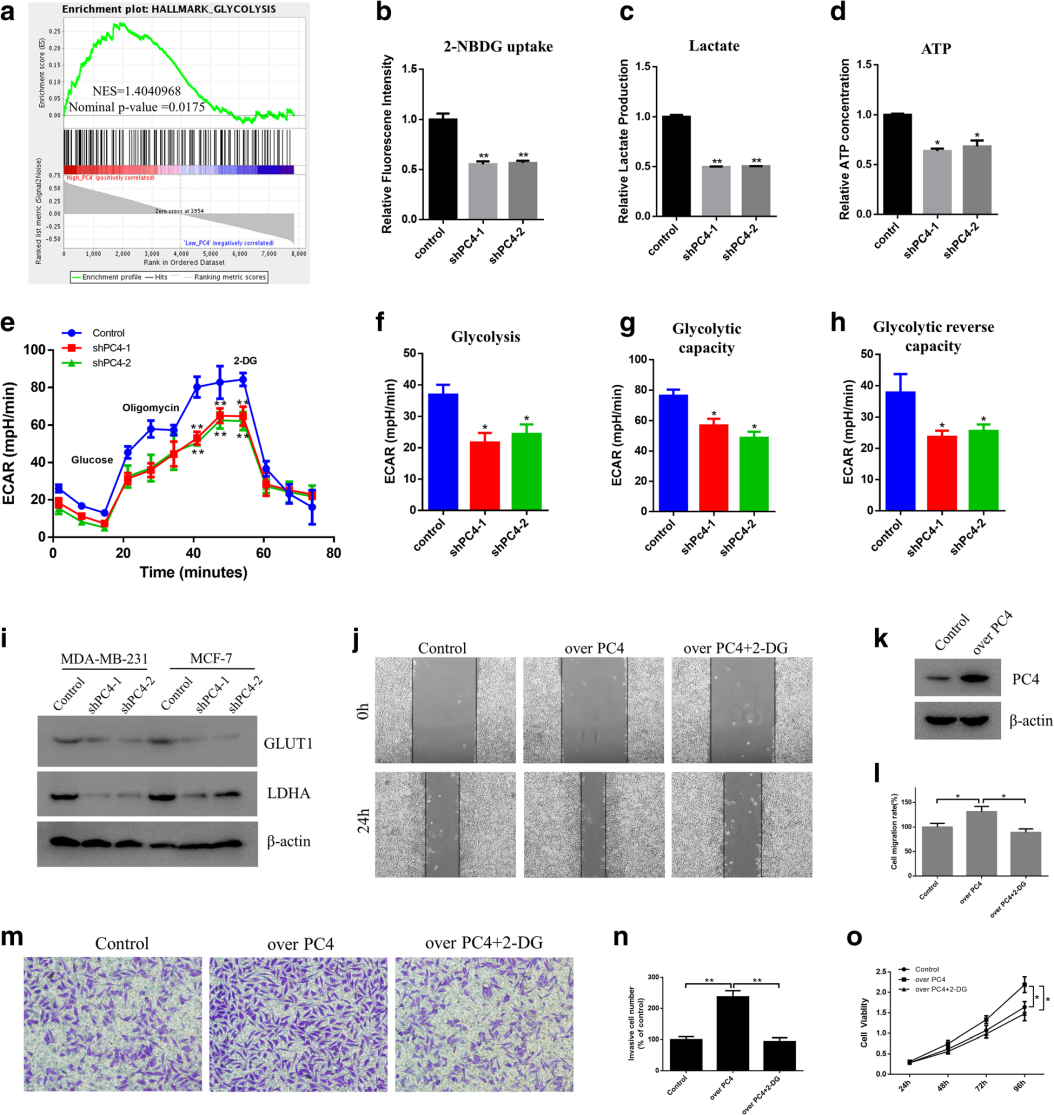
Glycolysis is critically involved in the oncogenic functional of PC4. **a** GSEA comparing the gene sets of glycolysis in PC4^high^ (n = 78) and PC4^low^ (n = 77) breast cancer patients. Data was obtained from GSE9893. NES means normalized enrichment score. **b, c** and **d** Glucose uptake, the production of lactate and ATP were determined in PC4 knockdown MDA-MB-231 cells and control cells. **e, f, g** and **h** The extracellular acidification rate (ECAR) was determined in PC4 knockdown MDA-MB-231 cells and control cells. **i** Glycolysis markers (GLUT1 and LDHA) were detected by western blotting in MDA-MB-231 and MCF-7 cells after PC4 knockdown. **j** Cell migration capacity was measured using the wound healing assay after 2-DG treatment in PC4 overexpression MDA-MB-231 cells. **k** The specific plasmid was used to overexpress of PC4 and the protein level of PC4 was examined by western blot. **l** Statistical analysis of the data derived from (j)**.** Experiments were repeated three times independently. **m** Cell invasive capacity was detected using the transwell assay in control cells, PC4 overexpression cells and 2-DG treated PC4 overexpression MDA-MB-231 cells. **n** Statistical analysis of the data derived from m**.** Experiments were repeated three times independently. **o** Cell viability was measured by CCK-8 assay in control cells, PC4 overexpression cells and 2-DG treated PC4 overexpression MDA-MB-231 cells. All data indicate the mean ± SD, *p < 0.05, **p < 0.01

To further illustrate the underlying mechanisms of PC4 mediated oncogenic advantages in breast cancer, GSEA in GSE9893 was conducted based on PC4 expression. As shown in Fig. 4a, the gene sets of HALLMARKS_GLYCOLYSIS were evidently enriched in PC4^high^ breast cancer samples (*P* < 0.05), indicating that glycolysis activity was positively correlated with high expression of PC4 in breast cancer. To our knowledge, Warburg effect, also known as aerobic glycolysis, is a remarkable hallmark of cancer, which provides the main source of energy and maintains the malignant phenotypes of cancer cells [46]. Thus, we tested whether PC4 could modulate the glycolytic phenotype in breast cancer cells. As expected, PC4 knockdown decreased glucose uptake (Fig. 4b), lactate production (Fig. 4c) and ATP generation (Fig. 4d) in MDA-MB-231 cells. PC4 knockdown also inhibited extracellular acidification rate (ECAR), which indicated overall glycolytic flux (Fig. 4e-h). Moreover, loss of PC4 inhibited the expression of GLUT1 and LDHA (Fig. 4i), the key enzymes of glycolysis. Hence, PC4 plays an essential role in maintaining Warburg effect in breast cancer cells.

Owing to the important role of Warburg effect in cancer development and progression [7], we next confirmed whether the oncogenic functions of PC4 were dependent on glycolysis. The glycolytic inhibitor 2-deoxy-D-glucose (2-DG) was used to block glycolysis in MDA-MB-231 cells, and the specific plasmid was used to overexpress PC4 before 2-DG treatment (Fig. 4k). Then, we found that overexpression of PC4 increased the proliferation (Fig. 4o), migration (Fig. 4j-l) and invasion (Fig. 4m-n) of breast cancer cells, which was inhibited by 2-DG. These results demonstrate that PC4 exerts its oncogenic functions by promoting glycolysis in breast cancer.

### PC4 promotes glycolysis by regulating c-Myc transcription

**Fig. 5:**
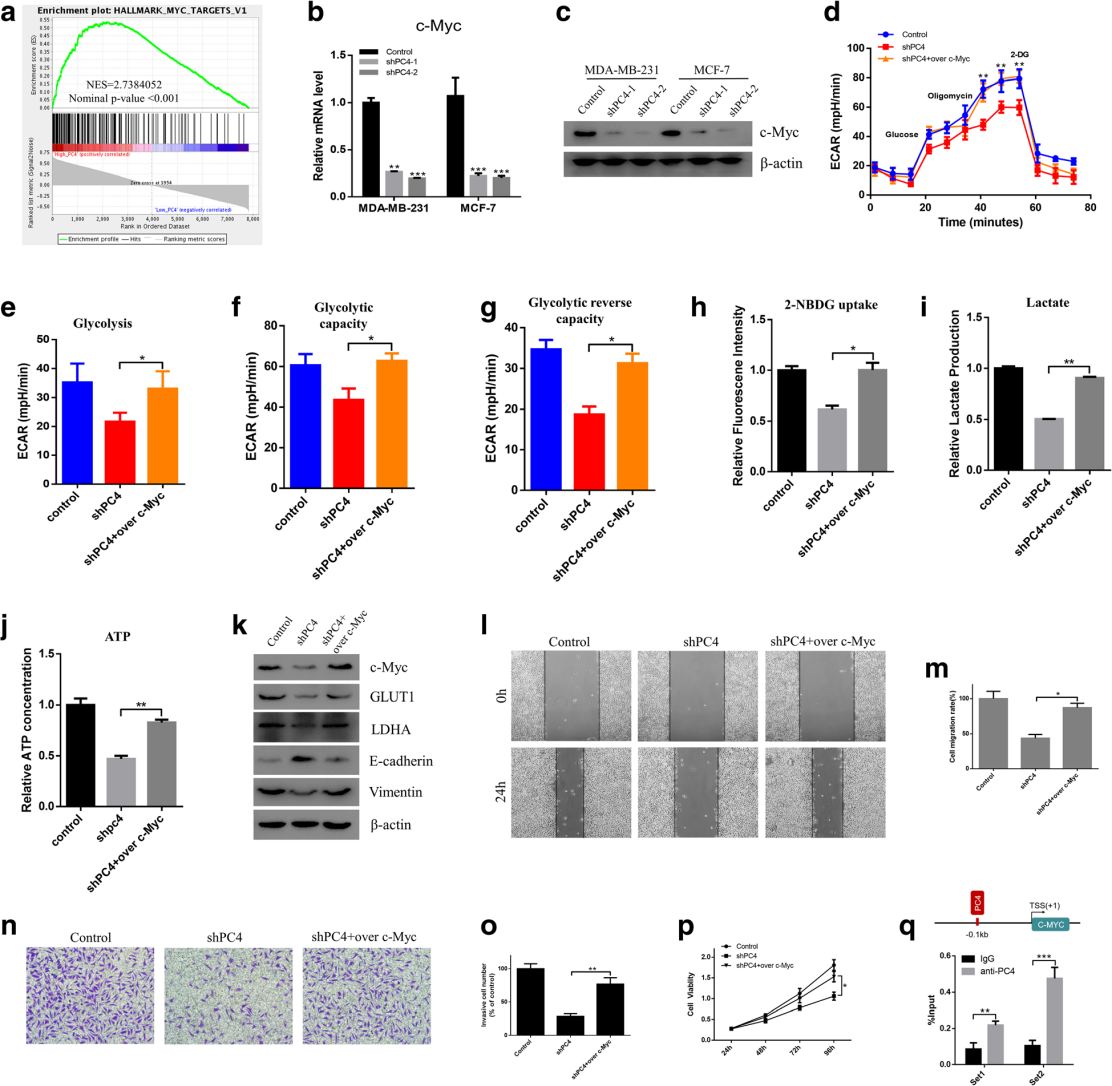
PC4 promotes glycolysis by regualting c-Myc transcription. **a** GSEA comparing the gene sets of Myc targets in PC4^high^ (n = 78) and PC4^low^ (n = 77) breast cancer patients. Data were obtained from GSE9893. NES means normalized enrichment score. **b** Protein levels of c-Myc were detected by western blotting in MDA-MB-231 and MCF-7 cells after PC4 knockdown. **c** mRNA levels of c-Myc were detected by qPCR in MDA-MB-231 and MCF-7 cells after PC4 knockdown. **d, e, f** and **g** After overexpression of c-Myc in PC4 knockdown MDA-MB-231 cells, the ECAR was measured. **h, i** and **j** After overexpression of c-Myc in PC4 knockdown MDA-MB-231 cells, glucose uptake, the production of lactate and ATP was measured. **k** After overexpression of c-Myc in PC4 knockdown MDA-MB-231 cells, the protein levels of c-Myc, GLUT1, LDHA, E-cadherin, and Vimentin were detected. **l** After overexpression of c-Myc in PC4 knockdown MDA-MB-231 cells, cell migration capacity was measured using the wound healing assay. **m** Statistical analysis of the data derived from l**.** Experiments were repeated three times independently. **n** After overexpression of c-Myc in PC4 knockdown MDA-MB-231 cells, cell invasive capacity was detected using the transwell assay. **o** Statistical analysis of the data derived from (n)**.** Experiments were repeated three times independently. **p** After overexpression of c-Myc in PC4 knockdown MDA-MB-231 cells, cell viability was measured by CCK-8 assay. **q** ChIP-PCR analysis for the PC4 occupancy on C-MYC promoters in MDA-MB-231 cells. All data indicate the mean ± SD. *p < 0.05, **p < 0.01, ***p < 0.001

Considering that c-Myc is a critical regulator of glycolysis in cancers by directly transactivating glycolytic genes [47], we performed GSEA in GSE9893 to explore the possible correlations between c-Myc and PC4. Interestingly, the gene sets of HALLMARKS_MYC_TARGETS_V1 were markedly enriched in PC4^high^ breast cancer samples (Fig. 5a**,** *P* < 0.001), indicating that c-Myc was positively correlated with high expression of PC4 in breast cancer. The qPCR (Fig. 5b) and western blot results (Fig. 5c) also showed that knockdown of PC4 observably downregulated the expression of c-Myc in both MDA-MB-231 and MCF-7 cells. Immunohistochemistry staining in nude mice xenografts (Additional file 1: Figure S3) also confirmed that loss of PC4 could decrease the expression of c-Myc in vivo. To further demonstrate whether c-Myc was responsible for the oncogenic functions of PC4, we used the specific plasmid to overexpress of c-Myc in PC4-knockdown cells (Fig. 5k). As expected, c-Myc overexpression rescued the decreased glucose uptake (Fig. 5h), lactate production (Fig. 5i) and ATP generation (Fig. 5j) in PC4-knockdown MDA-MB-231 cells. Overexpression of c-Myc also reversed extracellular acidification rate (ECAR) loss in PC4-knockdown cells (Fig. 5d-g). Moreover, the inhibitory effects of PC4 knockdown on proliferation (Fig. 5p), migration (Fig. 5l-m) and invasion (Fig. 5n-o) were also rescued by c-Myc overexpression. At last, the downregulation of GLUT1, LDHA, Vimentin and upregulation of E-cadherin in PC4-knockdown cells were reversed by overexpression of c-Myc (Fig. 5k). Collectively, these results suggest that PC4 exerts its oncogenic functions via activating c-Myc mediated glycolysis.

Given the important role of PC4 in transcription regulation [19], we subsequently examined whether PC4 could directly activate c-Myc transcription to promote its expression. To confirm the relationship between PC4 and c-Myc, we performed a bioinformatic analysis for the presence of putative PC4-binding sites (BS) in the C-MYC promoter, and found two putative PC4 BS in a region encompassing 0.1 kb upstream the transcription start site (TSS). Chromatin Immunoprecipitation (ChIP) showed that PC4 binds to the C-MYC promoter in that region (Fig. 5q), indicating that C-MYC is a direct downstream target of PC4.

## Discussion

Recurrence and metastasis are the two leading causes of death in breast cancer, especially in Oestrogen receptor (ER), progesterone receptor (PR) and human epidermal growth factor receptor 2 (HER2) triple-negative breast cancer (TNBC) [48]. Clarifying the underlying mechanisms of TNBC progression and developing novel therapeutic targets are crucial for breast cancer treatment [49, 50]. Here, we report a novel oncogene, PC4, which plays an important role in both ER-positive MCF-7 and triple-negative MDA-MB-231 breast cancer cell growth and metastasis by enhancing c-Myc mediated Warburg effect.

PC4 is initially identified as a transcription coactivator and contains highly conserved sequence among human, rat, mouse and yeast [12, 13]. As a nuclear protein, PC4 plays important roles in various cellular processes including basal transcription [14–17], DNA replication [18–22], DNA repair [23–31] and chromatin organization [32, 33]. More specifically, PC4 interacts with distinct domains of transcription activators such as GAL4-VP16 [51], AP2 [52] and SMYD3 [22] to regulate their functions. Based on the studies of its biological function, we have reported that PC4 was significantly upregulated in the malignant transformation of normal dermal multipotent fibroblasts, implying the crucial role of PC4 in tumorigenesis [35]. We and other researchers also showed that PC4 was highly expressed in several tumors [36–38], and regulated lymphatic metastasis [39] and chemo-radiosensitivity [26, 40, 41]. Although some studies showed the potential oncogenic role of PC4 in tumor, the moleculer mechanisms of PC4 in tumorigenesis and cancer progression are still unclear, especially in breast cancer. In this study, we specifically characterized PC4 expression in breast cancer. From immunohistochemistry results in both IDC and ILC, PC4 was demonstrated to be significantly upregulated and closely correlated with metastasis. The Kaplan-meier analysis of clinical data showed that PC4 was a potential predictor of poor prognosis in breast cancer. These findings led us to further explore the functional significance of increased PC4 expression in breast cancer. Through GSEA on 155 cases of breast cancer patients, we found that the gene sets of proliferation and EMT were positively correlated with high expression of PC4 in breast cancer. In both in vitro and in vivo experiments, PC4 knock-down obviously inhibited the growth and metastasis of breast cancer. Taken together, PC4 may be a promising therapeutic target in breast cancer.

As a hallmark of cancer cells, metabolic reprogramming is a key process during tumorigenesis and cancer progression [53]. Cancer cells frequently exhibit high rates of aerobic glycolysis, which facilitates cancer growth and progression by increasing glucose uptake, elevating lactate production, and supporting the energy demands [54]. In the process of glycolysis, c-Myc plays a key role by directly regulating the glycolytic enzyme genes [55]. By GSEA, we found that glycolysis-related genes and c-Myc target genes were positively correlated with high expression of PC4 in breast cancer. These findings led us to explore whether the oncogenic functions of PC4 were dependent on c-Myc mediated glycolysis. Indeed, PC4 knock-down dramatically inhibited the expression of c-Myc, as well as glucose uptake, lactate production, ATP generation, ECAR and the protein level of GLUT1 and LDHA. And Overexpression of c-Myc could reverse the decreased glycolysis and malignant phenotypes in PC4-knockdown cells, implying that PC4 exerted its oncogenic roles by c-Myc mediated glycolysis. Finally, the ChIP assays revealed that PC4 could directly bind to c-Myc promoters and transcriptionally activate c-Myc to promote its expression. Given the central role of c-Myc in many human cancers especially in triple negative breast cancer [56], c-Myc remains a promising target for effective antitumor therapy [57]. Unfortunately, c-Myc presents specific, significant obstacles to develop a strategy for its direct inhibition [57]. Thus, it is critical that the c-Myc transcription regulation be well-understood, particularly in its role in cancer progression and development.

## Conclusion

**Fig. 6:**
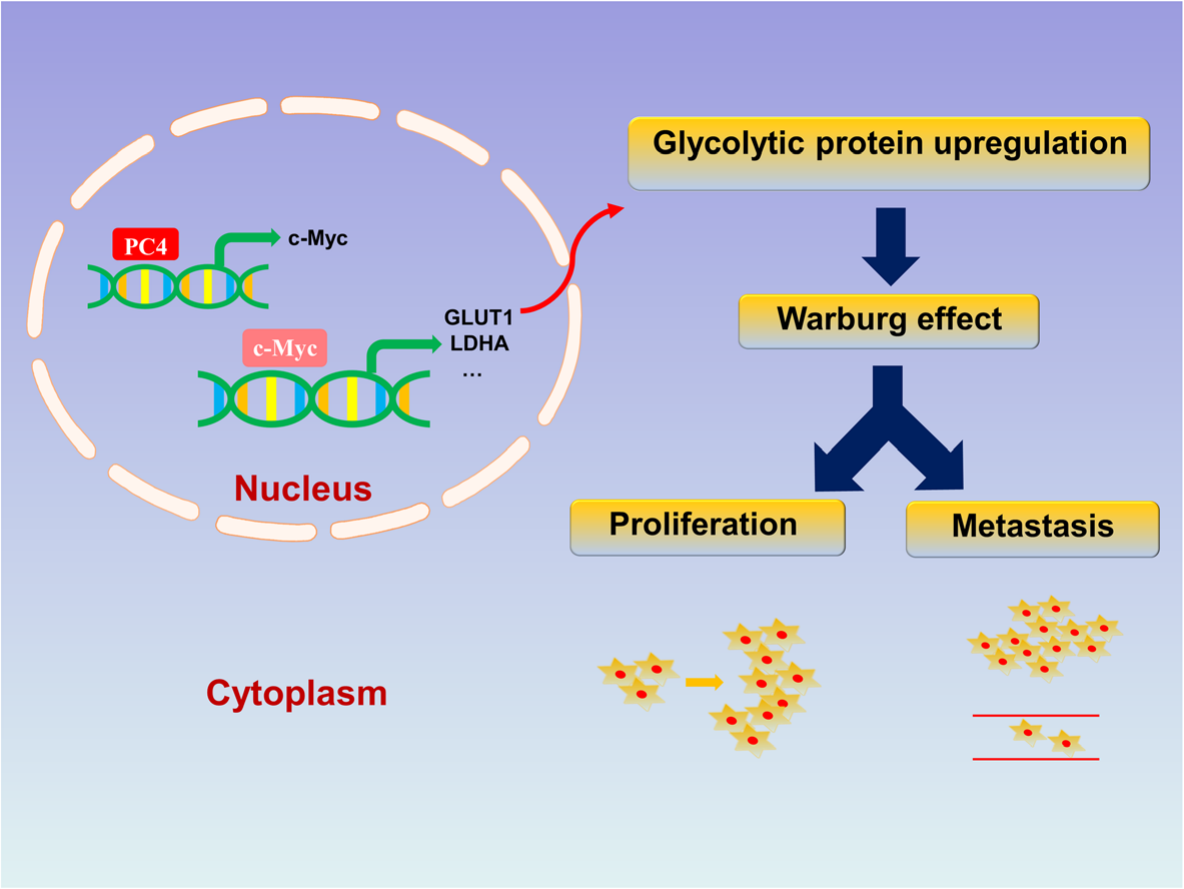
Possible Mechanism of PC4 mediated promotion of breast cancer proliferation and metastasis. Schematic representation of PC4 oncogenic functions in breast cancer cells. PC4 exerts its oncogenic functions by directly binding to c-Myc promoters and inducing Warburg effect to promote tumor growth and metastasis

This study is the first report of the expression pattern, diagnostic and prognostic value and oncogenic roles of PC4 in breast cancer. Furthermore, as a newly identified upstream regulator of c-Myc in breast cancer, PC4 exerts its oncogenic functions by directly binding to c-Myc promoters and inducing Warburg effect (Fig. 6). Therefore, our study provides novel insights into the functions and mechanisms of PC4 in breast cancer, and suggest that PC4 may be a novel therapeutic target for breast cancer.

## Additional file

Additional file 1: **Table S1** QPCR Primer sequences used, Related to the Methods. **Table S2.** QPCR Primer sequences used either for PC4 chromatin immunoprecipitation, related to the Methods. **Figure S1** Kaplan-Meier analysis for the association of PC4 expression (probe 212857_x_at and 214512_s_at) with overall survival time in breast cancer patients. Data was obtained using the Kaplan-Meier plotter. **Figure S2.** Silencing of PC4 have no significant impact on cell apoptosis in both MDA-MB-231 and MCF-7 cells. **Figure S3 a** MDA-MB-231 cells with stable PC4-knockdown were inoculated into female nude mice. The dissected xenografts were collected for immunohistochemical staining to detect the protein level of c-Myc, LDHA and Vimentin. **b** Statistical analysis of expression intensity derived from (a). Experiments were repeated three times independently. **p < 0.01, ***p < 0.001. (ZIP 17345 kb)

## Abbreviations

2-DG: 2-deoxy-D-glucose
CCK8: Cell counting kit-8
ChIP: Chromatin immunoprecipitation
ECAR: Extracellular acidification rate
EMT: Epithelial-Mesenchymal Transition
GSEA: Gene set enrichment analysis
IDC: invasive ductal carcinoma
ILC: Invasive lobular carcinoma
PC4: Positive cofactor 4
qPCR: quantitative polymerase chain reaction
