# Supplementary figures and images for: Transcriptional positive cofactor 4 promotes breast cancer proliferation and metastasis through c-Myc mediated Warburg effect
Source: Cell Commun Signal. 2019 Apr 16;17:36. doi: 10.1186/s12964-019-0348-0 (PMC6469038; doi:10.1186/s12964-019-0348-0)

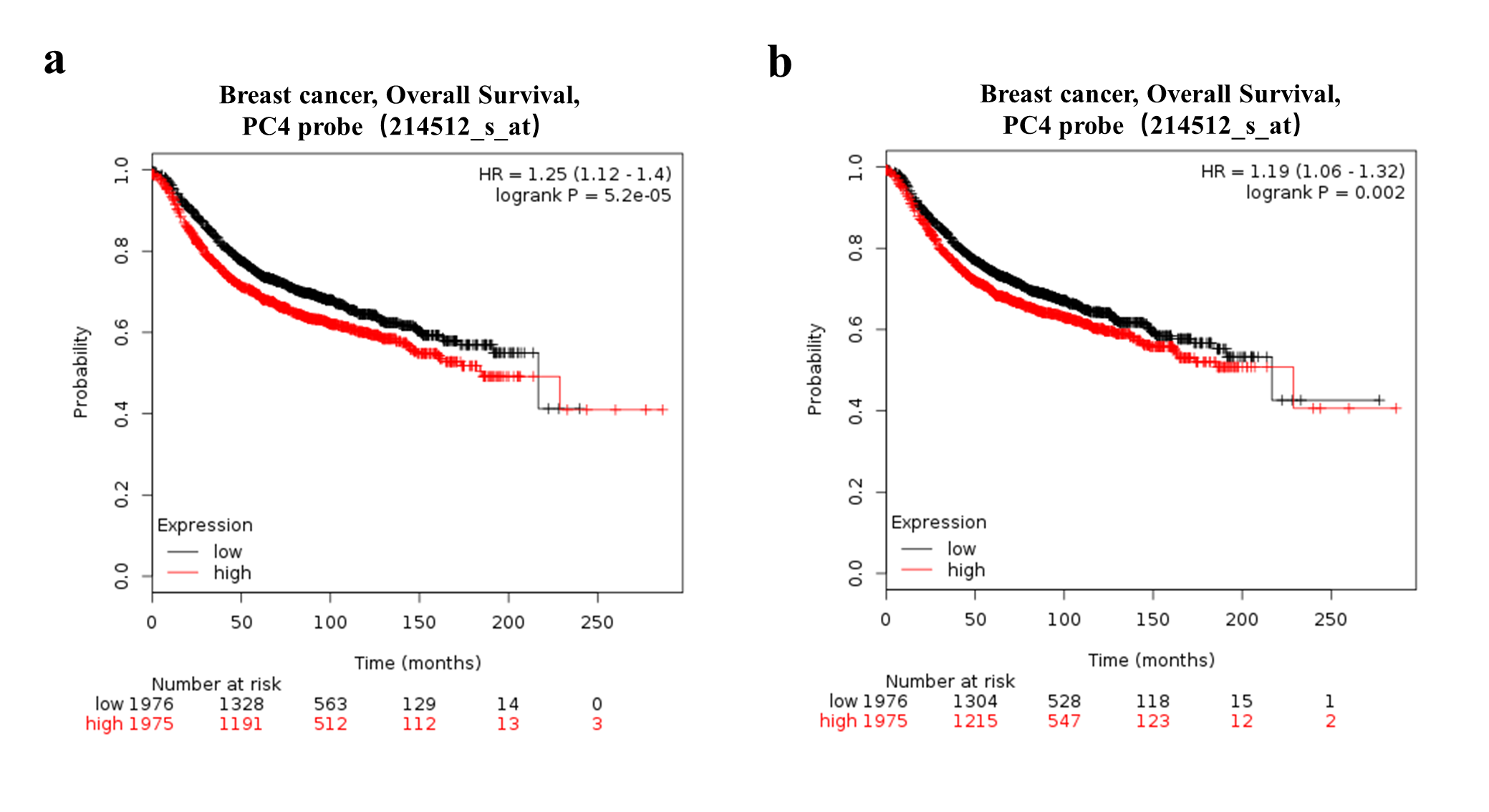

Supplement: Supplementary file 1 — Table S1 QPCR Primer sequences used, Related to the Methods. Table S2. QPCR Primer sequences used either for PC4 chromatin immunoprecipitation, related to the Methods. Figure S1 Kaplan-Meier analysis for the association of PC4 expression (probe 212857_x_at and 214512_s_at) with overall survival time in breast cancer patients. Data was obtained using the Kaplan-Meier plotter. Figure S2. Silencing of PC4 have no significant impact on cell apoptosis in both MDA-MB-231 and MCF-7 cells. Figure S3 a MDA-MB-231 cells with stable PC4-knockdown were inoculated into female nude mice. The dissected xenografts were collected for immunohistochemical staining to detect the protein level of c-Myc, LDHA and Vimentin. b Statistical analysis of expression intensity derived from (a). Experiments were repeated three times independently. **p < 0.01, ***p < 0.001. (ZIP 17345 kb) [file 12964_2019_348_MOESM1_ESM.zip › figS1.tif]

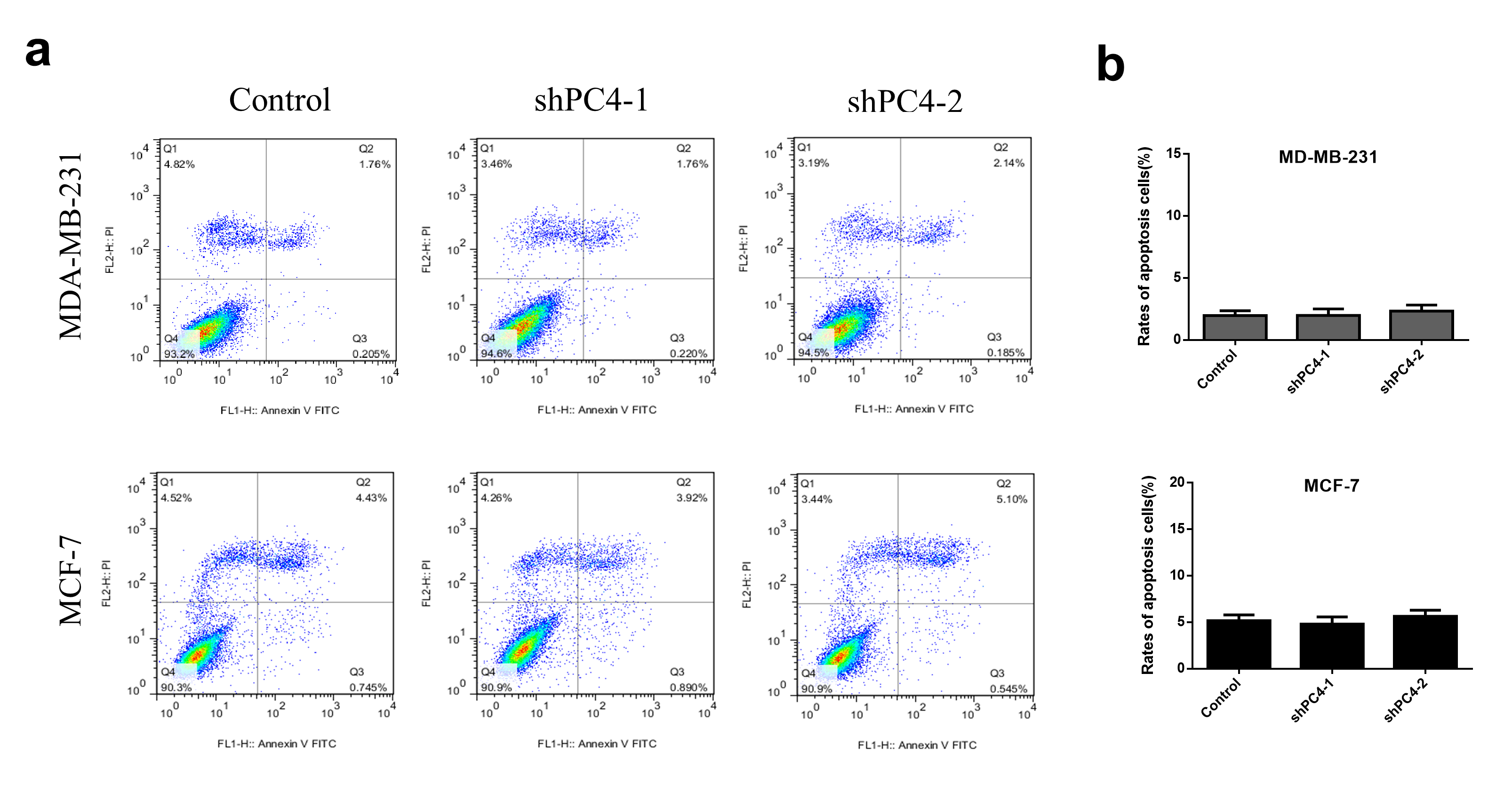

Supplement: Supplementary file 1 — Table S1 QPCR Primer sequences used, Related to the Methods. Table S2. QPCR Primer sequences used either for PC4 chromatin immunoprecipitation, related to the Methods. Figure S1 Kaplan-Meier analysis for the association of PC4 expression (probe 212857_x_at and 214512_s_at) with overall survival time in breast cancer patients. Data was obtained using the Kaplan-Meier plotter. Figure S2. Silencing of PC4 have no significant impact on cell apoptosis in both MDA-MB-231 and MCF-7 cells. Figure S3 a MDA-MB-231 cells with stable PC4-knockdown were inoculated into female nude mice. The dissected xenografts were collected for immunohistochemical staining to detect the protein level of c-Myc, LDHA and Vimentin. b Statistical analysis of expression intensity derived from (a). Experiments were repeated three times independently. **p < 0.01, ***p < 0.001. (ZIP 17345 kb) [file 12964_2019_348_MOESM1_ESM.zip › figS2.tif]

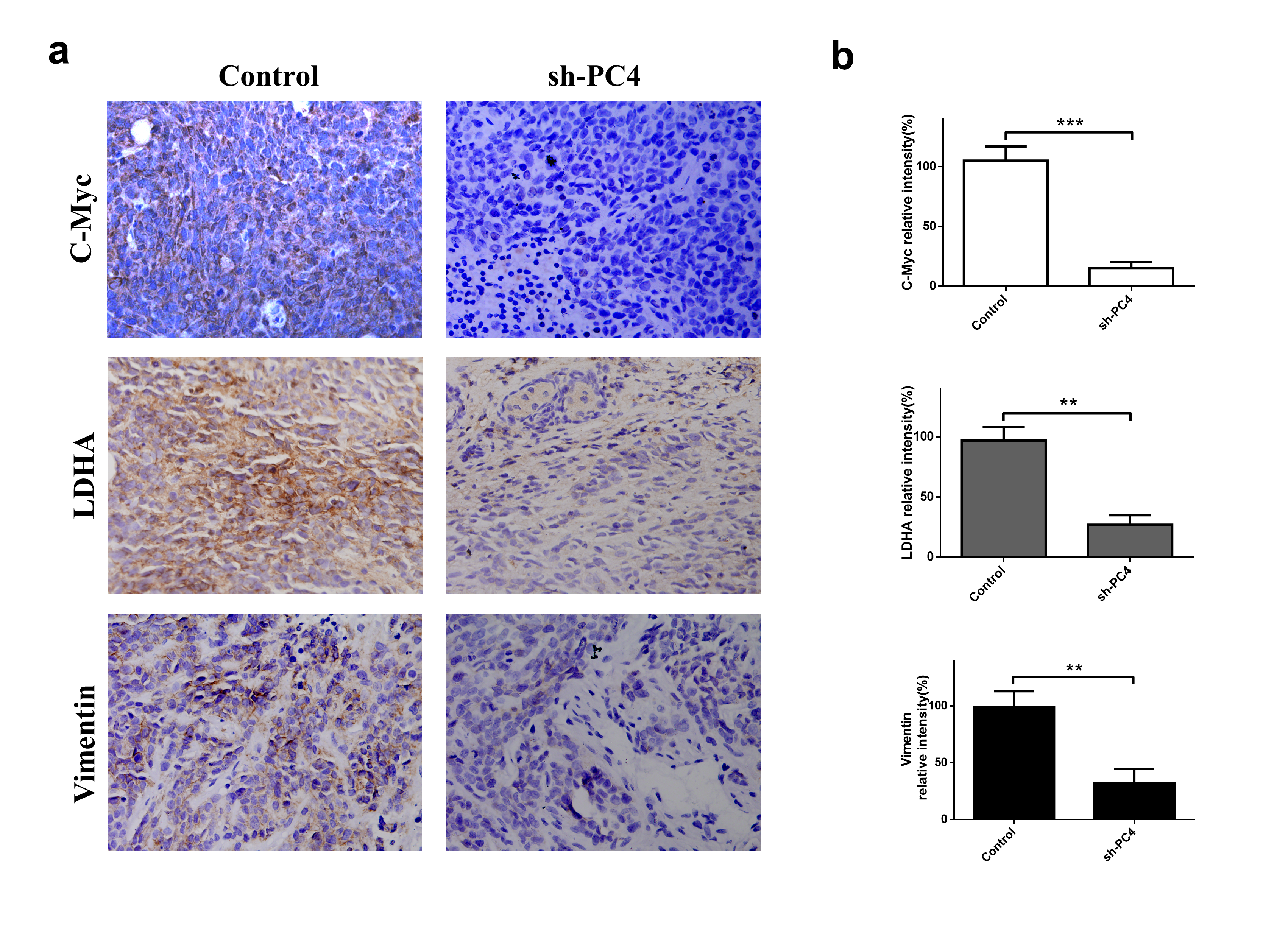

Supplement: Supplementary file 1 — Table S1 QPCR Primer sequences used, Related to the Methods. Table S2. QPCR Primer sequences used either for PC4 chromatin immunoprecipitation, related to the Methods. Figure S1 Kaplan-Meier analysis for the association of PC4 expression (probe 212857_x_at and 214512_s_at) with overall survival time in breast cancer patients. Data was obtained using the Kaplan-Meier plotter. Figure S2. Silencing of PC4 have no significant impact on cell apoptosis in both MDA-MB-231 and MCF-7 cells. Figure S3 a MDA-MB-231 cells with stable PC4-knockdown were inoculated into female nude mice. The dissected xenografts were collected for immunohistochemical staining to detect the protein level of c-Myc, LDHA and Vimentin. b Statistical analysis of expression intensity derived from (a). Experiments were repeated three times independently. **p < 0.01, ***p < 0.001. (ZIP 17345 kb) [file 12964_2019_348_MOESM1_ESM.zip › figS3.tif]
