# Supplementary material for: Transcriptional positive cofactor 4 promotes breast cancer proliferation and metastasis through c-Myc mediated Warburg effect
Source: Cell Commun Signal. 2019 Apr 16;17:36. doi: 10.1186/s12964-019-0348-0 (PMC6469038; doi:10.1186/s12964-019-0348-0)
Supplement: Supplementary file 1 — Table S1 QPCR Primer sequences used, Related to the Methods. Table S2. QPCR Primer sequences used either for PC4 chromatin immunoprecipitation, related to the Methods. Figure S1 Kaplan-Meier analysis for the association of PC4 expression (probe 212857_x_at and 214512_s_at) with overall survival time in breast cancer patients. Data was obtained using the Kaplan-Meier plotter. Figure S2. Silencing of PC4 have no significant impact on cell apoptosis in both MDA-MB-231 and MCF-7 cells. Figure S3 a MDA-MB-231 cells with stable PC4-knockdown were inoculated into female nude mice. The dissected xenografts were collected for immunohistochemical staining to detect the protein level of c-Myc, LDHA and Vimentin. b Statistical analysis of expression intensity derived from (a). Experiments were repeated three times independently. **p < 0.01, ***p < 0.001. (ZIP 17345 kb) [file 12964_2019_348_MOESM1_ESM.zip › Supporting Information-Revision.doc]

Supporting Information

**Transcriptional positive cofactor 4 promotes breast cancer proliferation and metastasis through c-Myc mediated Warburg effect**

Peng Luo^1†^, Chi Zhang^1†^, Fengying Liao^1^, Long Chen^1^, Zhenyu Liu^2^, Lei Long^1^, Zhongyong Jiang^1^, Yawei Wang^1^, Ziwen Wang^1^, Zujuan Liu^1^, Hongming Miao^3^ and Chunmeng Shi^1*^

* Correspondence: shicm@tmmu.edu.cn

†Equal contributors

^1^Institute of Rocket Force Medicine, State Key Laboratory of Trauma, Burns and Combined Injury, Third Military Medical University, Chongqing 400038, China

^2^Institute of Immunology, Third Military Medical University, Chongqing 400038, China

^3^Department of Biochemistry and Molecular Biology, Third Military Medical University, Chongqing 400038, China

Full list of author information is available at the end of the article

Supplementary Data

**Figure S1.** Kaplan-Meier analysis for the association of PC4 expression (probe 212857_x_at and 214512_s_at) with overall survival time in breast cancer patients. Data was obtained using the Kaplan-Meier plotter.

**Figure S2.** Silencing of PC4 have no significant impact on cell apoptosis in both MDA-MB-231 and MCF-7 cells.

**Figure S3.** **a** MDA-MB-231 cells with stable PC4-knockdown were inoculated into female nude mice. The dissected xenografts were collected for immunohistochemical staining to detect the protein level of c-Myc, LDHA and Vimentin. **b** Statistical analysis of expression intensity derived from (a). Experiments were repeated three times independently. **p<0.01, ***p<0.001.

**Table S1**. QPCR Primer sequences used, Related to the Methods.

| Gene | Forward | Reverse |
| --- | --- | --- |
| E-cadherin | CAGCCTGTCGAAGCAGGATT | CGCTCTCCTCCGAAGAAACA |
| N-cadherin | ATTGGACCATCACTCGGCTT | CACACTGGCAAACCTTCACG |
| Vimentin | GGACCAGCTAACCAACGACA | AAGGTCAAGACGTGCCAGAG |
| C-MYC | CAGCTGCTTAGACGCTGGATTT | ACCGAGTCGTAGTCGAGGTCAT |
| GAPDH | GGCATCCTGGGCTACACT | CCACCACCCTGTTGCTGT |

**Table S2**. QPCR Primer sequences used either for PC4 chromatin immunoprecipitation, related to the Methods.

| Gene | Primer name | Forward | Reverse |
| --- | --- | --- | --- |
| C-MYC | Site 1 | CCAACAAATGCAATGGGAGT | GGAGGAAAACGATGCCTAGA |
|  | Site 2 | CAGGAGGGGCGGTATCTG | TGTATTATGCATTATGTATGC ACAGC |
